# Why psychiatry needs an honest dose of gentle medicine

**DOI:** 10.3389/fpsyt.2023.1167910

**Published:** 2023-04-21

**Authors:** Lisa Cosgrove, Gianna D'Ambrozio, Farahdeba Herrawi, Moira Freeman, Allen Shaughnessy

**Affiliations:** ^1^Department of Counseling and School Psychology, University of Massachusetts Boston, Boston, MA, United States; ^2^School of Medicine, Tufts University, Boston, MA, United States

**Keywords:** gentle medicine, polypharmacy, conflicts of interest (COIs), psychotropic medications, pharmaceutical industry

## Abstract

The pharmaceutical industry’s influence on psychiatric research and practice has been profound and has resulted in exaggerated claims of the effectiveness of psychotropic medications and an under-reporting of harms. After the regulatory approval of fluoxetine, the pharmaceutical industry began promoting (and continues to promote) a chemical imbalance theory of emotional distress. In the last decade, there has been an increased awareness about the limits of this theory and the risks of psychotropic medications. Nonetheless, the medicalization of distress, the sedimented belief in “magic bullets,” and the push to “scale up” mental health treatment have contributed to the meteoric rise in the prescription of psychiatric drugs and of polypharmacy. A major premise of this paper is that the conceptual framework of medical nihilism can help researchers and clinicians understand and address the harms incurred by inflated claims of the efficacy of psychotropic medications. We propose that psychiatry, and the mental health field more generally, adopt a model of ‘gentle medicine’ with regard to both the diagnosis of and treatment for mental health conditions and focus greater attention on the upstream causes of distress.

## Introduction

All medical specialties have grappled with concerns about overdiagnosis, overtreatment, and the risk/benefit ratio of commonly prescribed medications. These concerns stem, in part, from the corrupting influence of academic-industry relationships on the evidence base. In fact, research conducted by scientists with ties to the pharmaceutical industry were 22 times less likely to report negative side effects than researchers without those ties (1, 2). Not surprisingly the underestimation and under-reporting of harms has led to medications being recalled for safety reasons. For example, two nonsteroidal anti-inflammatory drugs have been withdrawn from the market after data suppressed by their manufacturers was brought to light (3, 4).

Industry influence on psychiatric research and practice has been profound and has resulted in publications and news reports that exaggerate claims of effectiveness while minimizing harms. In 2003, the antidepressant nefazodone hydrochloride (Serzone) was withdrawn from the market because of documented concerns about hepatotoxicity (4), and antidepressants now come with a “black box warning” because of the trial data showing an increased risk of suicidality and other adverse events in youth (5). Le Noury and colleagues (6) conducted a re-analysis of SmithKline Beecham’s Study 329, an influential study concluding that paroxetine was safe and effective in adolescents. After obtaining access to the full unpublished dataset, Le Noury et al. (6) found an increase in harms for paroxetine that was not reported in the published literature. Specifically, the researchers concluded that paroxetine was associated with suicidal ideation and behavior other adverse events and that “access to primary data from trials has important implications for both clinical practice and research, including that published conclusions about efficacy and safety should not be read as authoritative” (6, p. 1).

These findings have led some to suggest there is both an intellectual and ethical crisis in mental health, (7, 8) one that continues to have significant public health consequences. The main aims of this paper are to (1) describe how this crisis developed; and (2) offer suggestions for more effectively responding to it. We draw on Stegenga’s (9) conceptual framework of medical nihilism in order to understand and address the harms incurred by inflated claims of the efficacy of psychotropic medications. We argue that the scientific and ethical crisis in psychiatry can only be ameliorated by the adoption of a model of ‘gentle medicine’ and by focusing attention on the upstream causes of distress.

### A crisis in the making: pharmaceutical marketing, neoliberalism, and the medicalization of distress

“It was definitely a clinical depression and one that I was going to have to have help to overcome. What I learned about it is your brain needs a certain amount of serotonin and when you run out of that, it’s like running out of gas, it’s like you’re on empty.”

-- Tipper Gore (10)

Where did former second lady of the United States Tipper Gore (10) “learn” that people need a certain amount of serotonin—just like a car needs gas to run? Of course, the answer to that question is complex, (11) but the marketing arms of the pharmaceutical companies that manufacture antidepressants played a key role in convincing the public about the magical properties of SSRIs, and concomitantly, serotonin. Psychiatrists Braslow and Marder recently summed this point up well, “From a cultural perspective, Prozac (fluoxetine; Eli Lilly and Company) has replaced Freud as shorthand for talking about what ails us” (11).

Indeed, shortly after fluoxetine (Prozac) came on the market it was generating over a billion USD per year, and soon accounted for a quarter of Eli Lilly’s profits (12, 13). As one journalist quipped in 2001 “Lilly is the house that Prozac built” (14). How much academic psychiatry explicitly contributed to the serotonin hypothesis and the “chemical imbalance” theory of depression and other mental health conditions is a matter of debate; Dawson and Pies (15) vehemently deny this suggestion. However, a recent systematic review of the literature on this topic (16) concluded that “the profession [of psychiatry] bears some responsibility for the propagation of [the serotonin theory of depression] and the mass antidepressant prescribing it has inspired.”

This much is certain: there are still leaders in the field who continue to promote various versions of the chemical imbalance theory and there has never been a public acknowledgement that this theory lacks scientific credibility. Also, after the regulatory approval of fluoxetine and other SSRIs, and the marketing and direct to consumer advertising campaigns that followed, the number of people taking antidepressants rose exponentially—and continues to rise. A 2020 Centers for Disease Control and Prevention (CDC) report found that during 2015–2018, 13.2% of adults were on antidepressant medications and more than twice as many women (17.7%) than men (8.4%) took them. Usage increased with age, particularly for women—use was found to be highest among women aged 60 and over (24.3%) (17). Additionally, global trends indicate that antidepressant use has risen in countries around the world, including China (18), the Netherlands (19), England (20), and Australia (21), to name a few.

It is important to note that the field organized itself around a biochemical theory of depression when SSRIs were being developed. When the DSM III and III-R were published in 1980 and 1987, the field officially adopted a medical model and hopes were high that the neurobiological basis of mental disorders would soon be known and concomitantly that drugs like fluoxetine would prove highly effective. These hopes were clearly evident when the president of the American Psychiatric Association announced in 1985, “our field is exploding with information, optimism, and enthusiasm. Psychiatry has moved from backwater to the forefront as a medical specialty, largely because of the research explosion, particularly in the neurosciences” (22).

Thus, in their zeal to achieve credibility the APA—and organized psychiatry more generally—quickly and enthusiastically embraced a specious scientific theory as medical reality, at great cost to society.

Of course, the exponential rise of psychotropic drugs, especially antidepressants, did not occur simply because of psychiatry’s need to “don the white coat” (i.e., be seen as a *bona fide* medical field) or because of pharmaceutical marketing (23). This rise also occurred in a political environment in which neoliberal capitalism led to both a lifting of marketing restrictions and a reduction in social supports provided by the government. In this environment, a new conceptualization of mental health developed, one that “responsibilizes” (24) people for their distress and obscures the connection between social injustice and emotional suffering. This new framework “led to income inequality, disempowerment of workers, inadequate social services, mass incarceration and an expensive and ineffective healthcare system” (25).

Not surprisingly, there is a burgeoning body of research demonstrating that neoliberal policies have likely contributed to the increasing rates of mental illness (25–27). The reasons are complex and multifaceted, but it is clear that neoliberal policies directly contribute to emotional distress *via* the consequences of precarity incurred by them. For example, a study of Indian farmer suicides (27), which have been rising and are among the highest in the world, found that neoliberal policies initiated an agrarian crisis and marginalized and destabilized small farmers. Specifically, the researchers found that cash crop production and high debt were strongly associated with suicide and recommended policy changes that would “stabilize the price of cash crops and relieve indebted farmers” as interventions that could reduce suicides (27).

Similarly, other researchers have shown that implementing more stringent requirements and punitive restrictions on welfare, coupled with the stigmatization of receiving benefits, likely led to the increase in suicides in the United Kingdom (28). Psychiatrist Helen Hansen (29) suggests that in the US, the increase in precarity incurred by the cuts to Social Security Income (SSI) may have led providers to medicalize distress; people who were no longer eligible for welfare and who needed assistance after social welfare programs were cut applied for SSI disability benefits, effectively “pathologizing the consequences of poverty in order to give their patients eligibility for financial assistance” (29). This medicalization of distress undermines an appreciation for the socio-political determinants of health and has undoubtedly contributed to the increase in the number of people diagnosed with psychiatric conditions and the increase in the prescription of psychotropic medications (and, as Hansen among others point out, “medication compliance” is often a requirement for receiving SSI benefits). It is also important to note that the DSM-III, by medicalizing distress and locating the causes of it inside the person rather than the toxic living conditions exacerbated by capitalism, inadvertently aided and abetted a neoliberal agenda. For example, the DSM-III’s “atheoretical” framework encouraged clinicians to diagnosis mental disorders in an acontextual way.

Additionally, in response to the movement for global mental health (MGMH), the World Health Organization (WHO) and other international organizations developed policies and programs whose aim is to ‘scale up’ mental health diagnosis and treatment, particularly in the global south. These policy initiatives and programs, while recognizing the importance of being responsive to local needs and culture, are based on Western biomedical conceptualizations of emotional distress. As such, they will likely lead to further overdiagnosis and overtreatment and deflect attention away from developing systemic and structural interventions.

For example, in one of the WHO campaigns, “Depression: Let us talk” (30), the focus is on increasing global awareness of depression: “When sadness does not stop: Helping Syrians talk about depression” (31). Although well-intentioned, this headline, and the larger campaign of which it is a part, reflects a neocolonial and neoliberal perspective. The assumption is that Western mental health interventions are best suited to remedy what the UN has described as the “biggest humanitarian and refugee crisis of our time” (32). In much the same way that in 1999 Tipper Gore (10) learned that she had depression as a result of her low serotonin, 20 years later we are teaching Syrians and other refugees and asylum seekers that their experience of violence and displacement is best understood as a psychiatric condition.

### The crisis continues: the rise of irrational polypharmacy and the continued search for magic bullets

Although there is no consensus definition of rational and irrational polypharmacy, over 20 years ago Kingsbury et al. (33) provided a helpful working definition. Rational polypharmacy refers to situations in which more than one medication is: 1) deemed clinically necessary to augment the effect of a drug, prevent a side effect, or treat comorbid conditions; (2) there is a clear body of evidence to support adding the medication; and, (3) the patient is closely monitored. Irrational polypharmacy, on the other hand, is when several of the same agents (e.g., second-generation antipsychotics, benzodiazepines, antidepressants) are prescribed because the patient is not responding to the first agent and the clinical focus is on treating each individual symptom rather than looking at the patient holistically (34, 35). The long-term effects of polypharmacy are unknown and there are little data to support its safety and efficacy, particularly in children and adolescents (34, 36–39). Also, for many patients it is difficult to taper or discontinue psychotropic medications and appropriate deprescribing protocols are only in the beginning stages of development. Factors that contribute to the growth of polypharmacy include the predominance of the biological model, erroneous assumptions about the efficacy of medication combinations, and limited knowledge of metabolic and neurological adverse drug events (40). Despite recognition that it increases the risk of adverse drug interactions and may create a cycle of using one drug to treat the adverse effects of another, irrational polypharmacy in psychiatry is rampant globally (41).

In fact, a study in the Netherlands (42), found that all the hospitalized adults with intellectual disabilities were polymedicated, primarily with antipsychotics and benzodiazepines. Moreover, 52% of drug prescriptions were classified as potentially inappropriate medications (PIM), “medications that should be avoided due to their risk which outweighs their benefit and when there are equally or more effective but lower risk alternatives are available” (42). Polypharmacy is common in older adults and psychotropic drugs are the most commonly prescribed medications, with questionable net benefit. In a study of adults 55 years and older in France, it was found that the threshold of two psychotropic medications increases the risk of impaired executive function, global cognition, and mobility, independent of confounding factors such as other comorbidities (43). In the United States, 13.18% of Medicare beneficiaries with a diagnosis of dementia were inappropriately prescribed second-generation antipsychotics (AP) for behavioral control and AP use was associated with higher inpatient visits, ER visits, and total costs (44). Globally, a meta-analysis found that the pooled prevalence of any antipsychotic use among people with dementia was 27.5% (45). International trends of AP use and prevalence has overall increased, with the global AP drug market expected to grow from 15.50 billion USD in 2022 to 24.74 billion USD in 2029 (46, 47).

Despite the growing body of literature demonstrating the harms associated with antidepressants and second-generation antipsychotics, and the recognition of the etiological complexity of mental health conditions, we continue to search for magic bullets, probably to our peril. The magic bullet concept was first put forth in the search for compounds that would effectively and selectively destroy bacterial cells without affecting animal cells. The hope for magic bullets currently drives medical research beyond curing infections. This hope is prominent in psychiatric research efforts searching for the single neurotransmitter or single type of neuroreceptor that, when triggered by an extraneous chemical—a pharmaceutical—will cure the patient of mental health afflictions as an infection is cured by an antibiotic. At its core, though, this thinking is akin to pointing to the use of an antipyretic to lower an infection-associated fever and pronouncing the patient infection free. As Ten Have and Gordjin (48) note, “The bizarre irony is that although magic bullets are rare, they are the driving force for many grandiose projects and enormous financial investments.”

For example, the recent suggestion that some people have “treatment resistant depression” (TRD), has led researchers to try to develop a one-size-fits-all intervention that will quickly and easily cure TRD. However, there is no consensually agreed upon definition of TRD (e.g., how many antidepressants must be tried or if psychotherapy or other interventions should be tried before applying the label), and no discussion about whether TRD is a valid construct. Perhaps, it is not the case that it is the depression that is resistant—perhaps, it is more accurate to acknowledge that antidepressants are not as effective as we originally hoped that they would be. Indeed, the infectious disease model is inappropriate; depression is not like a bacterial infection and we do not have strands of depression that are resistant to antidepressants.

Nonetheless, the United States Food and Drug Administration Safety (FDA) recently approved Janssen’s application for Spravato (Esketamine) through the agency’s breakthrough pathway designation. The FDA’s innovation Act (FDASIA) introduced “breakthrough therapy” in 2012 and has been used to justify applications for which the bar for regulatory approval is much lower because “a complete set of clinical data is not required” (49). In contrast, the National Institute for Health and Clinical Excellence (NICE) in the United Kingdom has recommended against its use (50).

When a regulatory bar is lowered, there are even less safety data required for approval. In fact, many of the new psychotropic drugs recently approved, *via* the ‘breakthrough designation pathway” or likely to be approved, may pose a significant risk of substance abuse and addiction (e.g., esketamine for “treatment-resistant depression” (51, 52); dextromethorphan HBr-bupropion HCI (Auvelity) for depression (53); and 3,4-Methylenedioxy methamphetamine (MDMA) in post-traumatic stress disorder” (54). The desire and search for a ‘magic bullet’ for mental health conditions paves the way for the rapid approval of drugs which lack robust evidence to support claims of efficacy and safety.

### Gentle medicine as a possible solution

“For many current scientific fields, claimed research findings may often be simply accurate measures of the prevailing bias.” (55)

As can be seen by this brief review, pharmaceutical marketing, guild interests, the biomedicalization of distress, and the concomitant failure to address the political etiologies of disorders (56) have created a perfect storm: the rise of psychotropics and irrational polypharmacy. Academic-industry relationships and publication bias against null findings have exacerbated the problem. The documented high placebo response and the growing awareness of side effects such as increased risk of self-harm, sexual dysfunction, and serotonergic syndrome for antidepressants, and weight gain, agranulocytosis, and increased risk of diabetes for second-generation antipsychotics have not dampened the appetite for “magic bullets.” This belief has fueled drug sales and contributed to psychiatry’s crisis of credibility.

Many researchers, clinicians, and policy-makers hoped that evidence-based medicine—using the data from randomized clinical trials and meta-analyses to inform clinical practice—would be the solution. Objectivity and engaging in evidence-based medicine are laudable goals, but the methods that are used for testing the effectiveness of medical interventions, as Stegenga (9) and others (48, 57, 58) have compellingly shown, are malleable. This malleability has resulted in a corruption of the scientific literature. Stegenga (9) provides many examples of failed medical interventions, including drugs that have been recalled, or labeled with black box warnings. He argues that “if we employ our best inductive framework, then our confidence in the effectiveness of medical interventions ought to be low,” and suggests that we engage in “medical nihilism”—a decidedly conservative approach to medicine and medical research. He critiques health care practitioners and medical researchers for their “magic bullet” reductionism that oversimplifies both health and illness and advocates for less aggressive, gentler medicine and a greater focus on non-medical interventions and care (9).

Stegenga advocates for an approach he calls “gentle medicine,” a proposal that physicians should intervene less and instead try to improve health with changes to patients’ lives and our society (9, p. 187). This suggestion is consistent with the founding impulse of modern medicine. Well over a century ago Oliver Wendell Holmes stated, “I firmly believe that if the whole materia medica as now used could be sunk to the bottom of the sea, it would be all the better for mankind—and all the worse for the fishes.” Nonetheless, at first glance it may seem unduly optimistic—even naïve—to talk about gentle medicine in an age of Big Pharma; after all, pharmaceutical companies are some of the most profitable in the world (59). However, this “when in doubt, do not” approach, is a type of “Hippocratic approach” in that it acknowledges the potential for harm with any medical intervention and emphasizes the need for stronger proof that benefits outweigh risks. Stegenga thus suggests that clinicians back away from a relatively uncritical approach to acceptance of new treatments and instead privilege treatment approaches that may have less evidence of effectiveness but more demonstration of safety. At its core though, the argument for gentle medicine is the conceptualization that we need more non-medical tools to address mental suffering.

Although Stegenga’s (9) critique is aimed at medicine in general, his point that there is a systematic bias in medical thinking that results in an overestimation of benefits and underestimation of harms is particularly relevant to psychiatry. Organized psychiatry, with some important exceptions (60–62), has been reluctant to acknowledge that this bias exists. For example, as recently as 2015, the American Psychiatric Association recommended antidepressants and electroconvulsive shock therapy (ECT) for even mild depression despite evidence demonstrating that both antidepressants and ECT do more harm than good for mild depression (63). Indeed, psychiatric interventions have often been developed through the use of inappropriate comparators, violating the principle of equipoise. These interventions are promoted by *post hoc* justification (64), furthering guild interests. Some have suggested that researchers publicly register their hypotheses before collecting data, “so as to prevent a-posteriori modifications that skew results” (1).

Thus, if psychiatry is to take the idea of gentle medicine seriously, the field would need to acknowledge the lack of effectiveness of many psychotropics, acknowledge their harms, and embrace a tolerance for uncertainty, while improving the lives of some people, these agents are simply not the magic bullets we hoped they would be. For example, organized psychiatry could adopt a robust person-centered and harm reduction model to psychotropics. Such an approach would shift the focus from symptom reduction and the assumption that all people diagnosed with psychiatric conditions require life-long medication toward a genuine appreciation for an individual’s unique life circumstances (65, 66). In the clinic, this would mean stopping the practice of adding a new drug when the current medication was not bringing the patient noticeable improvement and instead pivoting to other modes of (non-drug) treatment. It also means taking research findings—ones that run counter to the dominant narrative—seriously, such as the recent meta-analysis which found that only about 15% of treated participants experience a substantial antidepressant effect beyond the placebo effect (67).

Indeed, if the field of psychiatry were to practice medical nihilism, there would be a willingness to question whether “treatment resistant depression” is a misnomer; the problem may very well be in our treatments rather than in the patient. Recognizing and accepting the limits of the effectiveness of medical interventions is not only a way to achieve epistemic and clinical humility, but also a way to direct attention to the political and structural causes of emotional distress. It is likely that if psychiatry and the mental health field more generally were to practice a gentler form of medicine, one that shifts the focus away from overdiagnosis and overtreatment and toward structural and systemic interventions, we will see greater gains in well-being at the population level. Unfortunately, the suggestion that the field take seriously research findings that run counter to the dominant narrative and address the socio-political causes of emotional suffering, is a tall order. Too frequently, cogent criticisms get deflected and marginalized and the critics are dismissed as being anti-psychiatry and anti-medication.

## Conclusion

“The expectation that drugs can intervene on one or a few micro physiological targets and thereby bring about an effect which is both clinically significant and symptomatically specific is, for many of our medical interventions, unfounded.” (9, p. 15).

After more than 40 years of trying to gain legitimacy in the medical community by adopting a medical model that conceptualizes all forms of emotional distress as “brain disorders” (68), there has been little improvement in mental health care. It is noteworthy that leaders within psychiatry (8, 69, 70) are saying the emperor has no clothes and are pushing for radically new solutions. The call for a paradigm shift was made dramatically by Tom Insel, MD; in a statement reviewing his tenure as head of the National Institute of Mental Health, he wrote, “…I think I succeeded at getting lots of really cool papers published by cool scientists at fairly large costs—I think $20 billion—I do not think we moved the needle in reducing suicide, reducing hospitalizations, improving recovery for the tens of millions of people who have mental illness” (71). In a similar vein, Alan Frances, MD, Chair of the DSM-IV, stated, “I object to the National Institute of Mental Health (NIMH) research agenda that is narrowly brain reductionistic; it has achieved great intellectual masterpieces, but so far has not yet helped a single patient” (69).

Clearly, the time is right for a reconceptualization of how to approach mental health and mental illness. The COVID-19 pandemic has been a major disruptor by forcing structural changes—ones that made us question seemingly self-evident truths (1). For example, work previously performed in offices can be effectively performed at home. Medical care need not always be provided face-to-face but can be delivered virtually. Indeed, COVID-19 has given us an opportunity to critically evaluate the way medical research and medical interventions are carried out and to think creatively about what a gentle medicine model would look like in psychiatry and in the mental health field more generally. It should be emphasized that gentle medicine should not be conflated with complete medical nihilism. Although research about drug treatments must continue, gentle medicine would mean abandoning the search for magic bullets and focusing more on the root causes—structural inequities in society—that play a major role in the development of anxiety, hopelessness, sorrow, dejection, despondency, emptiness, and despair (2). As the former United Nations Special Rapporteur on the Right to Health eloquently stated, “Mental health and well-being cannot be defined by the absence of a mental health condition, but must be defined instead by the social, psychosocial, political, economic, and physical environment that enables individuals and populations to live a life of dignity, with full enjoyment of their rights and in the equitable pursuit of their potential” (72).

For psychiatry and related fields to adopt this gentle medicine approach, changes would need to occur at multiple levels and there must be an openness to learn from other specialties that have adopted a less-is-better approach. The specialty of geriatrics is often described as, “taking patients off of medicines they do not need.” Similarly, the specialty of family medicine aims to minimize unnecessary health care and has made strides in addressing the health-harming legal needs (e.g., precarious/unsafe housing; denial of food benefits; obstacles in the pathways to citizenship), of patients (73). At the conceptual level, it is time to revisit psychotherapy theories and modalities, ones that emphasize the critical importance of attending to context, meaning-making, and the social determinants of health (these theories and concepts got short shrift with the move to a medical model). On the macro level, there needs to be an acknowledgment that our trust in medicine in general and in psychiatric interventions in particular is disproportionate (1) and there needs to be greater appreciation for the ways in which inequity is health-harming. The structural competency movement (74) in psychiatry, which seeks to understand the relationships among race, class, and symptoms and *acts on* systemic causes of health inequalities, is an excellent example of what a paradigm shift might look like.

Indeed, psychiatrists and mental health clinicians need to join their colleagues in other specialties to advocate for social change. Relatedly, there needs to be a restructuring of medical education by de-emphasizing the training of psychiatrists-as-psychopharmacologists and by developing a workforce of activist therapists to augment psychiatric gentle medicine. Last, there needs to be injected into everyday practice a healthy dose of epistemic and intellectual humility through “honest self-disclosure, avoidance of arrogance, and modulation of self-interest” (75).

## Author contributions

LC conceptualized the paper, along with AS, and developed the first draft of the paper and reviewed and contributed to all subsequent drafts. AS drafted the section on medical nihilism and the conclusion and contributed to all sections of the paper. GD'A drafted the section on psychotropic polypharmacy and MF conducted literature searches, complied background searches, and contributed to the section on medical nihilism. FH drafted the section on the FDA’s regulatory pathways, conducted background research, and contributed to other sections. All authors contributed to manuscript revision and read and approved the submitted version.

## Conflict of interest

The authors declare that the research was conducted in the absence of any commercial or financial relationships that could be construed as a potential conflict of interest.

## Publisher’s note

All claims expressed in this article are solely those of the authors and do not necessarily represent those of their affiliated organizations, or those of the publisher, the editors and the reviewers. Any product that may be evaluated in this article, or claim that may be made by its manufacturer, is not guaranteed or endorsed by the publisher.
